# Ethnoveterinary plant remedies used by Nu people in NW Yunnan of China

**DOI:** 10.1186/1746-4269-6-24

**Published:** 2010-08-26

**Authors:** Shicai Shen, Jie Qian, Jian Ren

**Affiliations:** 1 Center for Biodiversity and Indigenous Knowledge, Kunming Yunnan 650034, PR China; 2 Kunming Institute of Botany, Chinese Academy of Sciences, Kunming 650204, PR China; 3 Pratacultural Science Department, Yunnan Agriculture University, Kunming 650201, PR China

## Abstract

**Background:**

Nu people are the least populous ethnic group in Yunnan Province of China and most are distributed in Gongshan County, NW Yunnan. Animal production plays an important role in Nu livelihoods and the Nu people have abundant traditional knowledge of animal management and ethnoveterinary practices. This study documents the animal diseases, ethnoveterinary plant remedies and related traditional knowledge in three Nu villages of Gongshan County.

**Methods:**

This study was carried out in three Nu villages of Gongshan County between July 2009 and February 2010. Data was obtained through the use of semi-structured questionnaires, field observation and PRA tools. A total of 60 Nu respondents (34 men and 26 women) provided information on animal ailments and ethnoveterinary plant medicines used for Nu livestock production. Information on traditional ethnoveterinary medicine knowledge and choice of treatment providers was also obtained.

**Results:**

Thirty-five animal conditions were identified in the surveyed area. The major and most common animal diseases among livestock were skin conditions, diarrhea, heat, fevers, colds, and parasites. Most ailments occurred between June and August. The ethnoveterinary medicinal use of 45 plant species was documented. Most medicinal species (86.7%) were collected from the wild. The most frequently used plant parts were whole plants (35.6%), followed by roots (22.2%). The most important medicinal plant species were *Saussurea costus *(Falc.) Lipech. (UV = 0.67), *Senecio scandens *Buch.-Ham.ex D.Don (UV = 0.67), *Plantago depressa *Willd. (UV = 0.63), *Rubus corchorifolius *L. f. (UV = 0.62), *Bupleurum yunnanense *Franch. (UV = 0.60), and *Polygonum paleaceum *Wall. (UV = 0.60). Animal diseases treated with the highest number of ethnoveterinary plant remedies were diarrhea (16 plant species), heat, fever, colds (11 plant species), retained afterbirth (11 plant species), and skin conditions and sores (11 plant species). Many Nu villagers (52%) considered traditional remedies their first choice of animal disease treatment. Traditional ethnoveterinary knowledge was related to the local social-cultural characteristics of Nu people and communities.

**Conclusion:**

Animal production plays an important role in Nu culture and livelihoods, and the Nu ethnic group has abundant traditional knowledge about animal production and ethnoveterinary plant remedies. This traditional knowledge faces the risk of disappearing due to increasing modern veterinary medicine extension, livelihood changes and environment degradation. Animal diseases are a major constraint in livestock production in Nu villages. Thus, some strategies and measures should be adopted in the future, such as further researches on Nu culture and livelihoods, community-based validation of ethnoveterinary medicine and broad network building and knowledge sharing.

## Background

Ethnoveterinary medicine, the scientific term for traditional animal health care, provides low-cost alternatives to allopathic drugs. Research into ethnoveterinary medicine is often undertaken as part of a community-based approach that serves to improve animal health and provide basic veterinary services in rural areas [1]. In addition to its focus on botanicals, ethnoveterinary medicine covers people's knowledge, skills, methods, practices, and beliefs about the care of their animals [2-5].

Ethnoveterinary medicine is frequently used for treating animal as well as human diseases by many different people around the world. According to the World Health Organization, at least 80% of people in developing countries depend largely on indigenous practices for the control and treatment of various diseases affecting both human beings and their animals. Ethnoveterinary medicine provides valuable alternatives to and complements western-style veterinary medicine. Ethnoveterinary remedies are accessible and easy to prepare and administer, at little or no cost to the farmer [6]. In many poor rural areas, ethnoveterinary medicine can play an important role in animal production and livelihood development, and often becomes the only available means for farmers treat ill animals [2-6].

Ethnoveterinary medicine and knowledge are an integrated part of Nu ethnic group culture and livelihoods and widely practiced in Nu communities in Yunnan Province, southwest China [7,8]. The Nu ethnic group is one of the least populous ethnic groups and earliest indigenous peoples [8] in Yunnan, primarily residing in Gongshan County. There are 7,142 Nu people, most of whom live in Dimaluo, Qiunatong and Shuangla administrative villages, Gongshan County [9]. The livelihood of Nu communities is typically agro-pastoralist and dependent on animal production. There are a great variety of animals reared by Nu people, many of which are indigenous species. Animals have various functions and make various contributions to Nu life and culture, both economic and social, such as exchange, wedding, funeral and religious functions [10]. For example, pigs are frequently used for human consumption, manure accumulation and exchange. The primary functions of chicken are human consumption and exchange. Cattle are often used for land plowing, manure accumulation and bank savings. Sheep/goats are used for bank savings. In general, the social functions of animals are more important than their economic functions for local Nu communities [10]. Due to cultural differences with other ethnic groups, Nu people have a great variety of unique traditional knowledge about livestock production and management and natural resource use. However, there is scant literature on the animal production, diseases, ethnoveterinary remedies, and ethnoveterinary knowledge of the Nu people.

This study has examined the illness conditions of livestock, ethnoveterinary plant remedies used in treating illnesses, ethnoveterinary medicinal knowledge, and choice of treatment providers among three Nu ethnic villages in Gongshan: Dimaluo, Qiunatong and Shuangla. The survey findings are important to understand the culture and livelihood patterns of Nu people and promote ethnoveterinary practices to use for animal health service in this poor ethnic minority area.

## Materials and methods

### Study area

**Figure 1:**
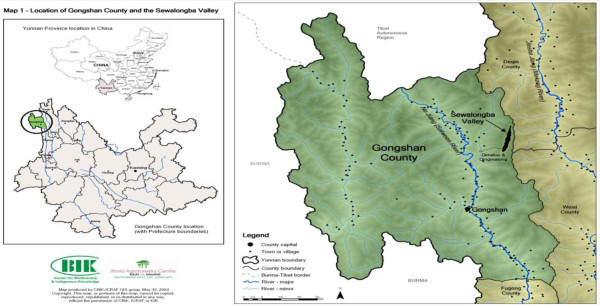
**Study area - Gongshan County, NW Yunnan of China (This picture was provided by CBIK and ICRAF)**.

The present study was undertaken in Gongshan County, NW Yunnan of China. The County is situated in the northwest end of Yunnan province, between 98° 08' - 98° 56'E and 27° 29' - 28° 23'N. To the west it borders Myanmar, to the north it borders Chawo County in Tibet, and to the east shares borders with Weixi County and Deqin County in Yunnan province (Figure 1). The Nujiang (Salween River) runs through the county roughly from north to south. With an elevation from 1,170 m to 5,128 m, the typical climate is characterized by both raining season of heavy rainfall with 90% humidity and dry season of few rains with drought. The rainfall is about 2,700-4,700 mm per year. Gongshan is a national level poverty county according to state criteria, with a mountainous area topography and mix of 15 ethnic groups, with the minority groups representing 96% of the total population [9]. The Dulong and Nu people of Gongshan are among the least populous ethnic groups in Yunnan.

The total area of Gongshan County is 4,506 sq. km with 4,437.3 hectares of arable land, 177,706.7 hectares of forested land, and 112,986.7 hectares of grassland [9]. Gongshan is a typical agro-pastoralist livelihood area of northwest Yunnan province [10]. Crop production and livestock are the major sources of cash income generation. There is a variety of livestock types and species in Gongshan including cattle, pig, goat/sheep, chicken, horse, and donkey. Some of these were introduced from outside by the Gongshan Animal Husbandry Bureau and private sector, but most are local indigenous species. Total livestock herd holdings were 64,936 in 2009, of which cattle and horse accounted for 14.9%, goats/sheep accounted for 37%, and pigs accounted for 48.1% [9].

### Methods

The main objectives of the study were to explore the traditional knowledge of animal illnesses and ethnoveterinary practices for livestock illness treatment in three Nu ethnic villages of Gongshan. The study area focused on three main Nu ethnic villages in Gongshan: Dimaluo, Diunatong and Shuangla.

Field data collection was undertaken from July 2009 to February 2010. Information on respondent characteristics, livestock illnesses, use of ethnoveterinary plant remedies used in treating illnesses, ethnoveterinary medicinal knowledge, and choice of treatment providers was obtained through semi-structured questionnaires complemented by free interviews and informal conversation [11,12]. In addition, Participatory Rural Appraisal (PRA) [13-18] and ethnobotanical and anthropological methods [19,20] were used to facilitate participation of local villagers in extraction and analysis of disease signs and symptoms, disease causes and solutions, plant distribution, and related traditional veterinary knowledge in the field. Interviews were carried out individually and collectively taking into consideration gender and age differences. Household samples were selected at random in three Nu ethnic villages of 20 Nu households in each village. Medicinal plants were collected, preserved, and then identified at the Yunnan Agriculture University. All primary data was entered in Excel and summarized into means and frequencies using SPSS 12.0.1 for windows. To compare the relative importance of each remedy practice, their use-values were calculated (adapted from the proposal of Phillips et al.) [21] using the following formula: UV = ΣU/n, where: UV is the use-value of a species; U is the number of citations of that species; and n is the number of informants. The use-value of each species is based only on the importance attributed by each informant and does not depend on the opinion of the researcher or the interviewees.

## Results and discussion

### Respondents' biographic details

The total respondents interviewed were 60: 20 from Dimaluo village (Pengdang Township), 20 from Qiunatong village (Binzhongluo Township) and 20 from Shuangla village (Binzhongluo Township). All respondents were Nu ethnic people. Thirty-four of the respondents were male and the rest female. The respondent age range was from 35 to 79 years old, with an average age of 51.3. Those people who were older had more experience and traditional knowledge of animal production. Most local farmers have converted to Catholicism and go to pray in Church on Sunday. The education level is poor overall. Young people have higher education than elders and females have lower education than males (Table 1).

**Table 1:** Demographic characteristics of the respondent

Characteristic	Frequency
Religion	
Catholicism	40
Buddhism	7
Christianism	13
Formal education	
Illiterate	15
Primary	24
Secondary	19
High and higher	2
Main occupation	
Farming	55
Salary work	1
Labour force	2
Trading	2
Gender	
Male	34
Female	26

Crop growing and livestock rearing (54.3% of total household income) were the main source of Nu livelihoods. Livestock reared by Nu households include pigs, chickens, goats, sheep, cattle and a few horses. Pigs and chickens are the livestock most commonly reared, followed by cattle and sheep. Only a few people raised horses. Livestock and crop growing have a close linkage and interaction in the Nu agriculture system. The main use of crops (76.1% of total yield) was for livestock fodder and a lower percentage was used for human consumption (16%). Animals provided manure/compost for crop growing.

### Common illness conditions among livestock

Surveys found that disease was a major cause of mortality among livestock in the three Nu ethnic villages. The death rates of pigs (24.2%) and chickens (29.8%) were higher in comparison to cattle (15.2%) and goats/sheep (15.2%) in 2009. Piglets and chicks often die in the spring and winter due to poor housing quality and climate conditions. Disease not only results directly in economic losses of livestock, but also requires Nu villagers to spend cash to recover livestock holdings and sometimes villagers even have to change their livelihood strategies. Like other communities in Gongshan County, disease was a major factor causing animal mortality and constraining the development of animal husbandry [9]. The morbidity and death rates of animals in the three Nu villages was found to be lower than other communities in Gongshan, however. One reason is these villages are far away from towns and markets; another reason is that Nu farmers often use ethnoveterinary medicines for animal illness treatment.

Though more than 35 different illness types were recorded as animal health problems in the three Nu villages, the most common major diseases according to respondents included skin conditions, diarrhea, heat, fevers, and, colds, and parasites. Gaseous stomachs and constipation, breathlessness, twitching, shivering, and frequent lying down were less common (Table 2). Most respondents had diagnosis knowledge of these diseases and could readily distinguish them on the basis of accepted signs and symptoms. For pigs, the most common diseases were skin conditions (81.7%), parasites (68.3), diarrhea (56.7%), and heat, fever, colds (55.8%). For chickens, diarrhea (66.7%), heat, fever, colds (81.7%) and Newcastle disease (83.3%) were more common. Diarrhea (65%) and heat, fever, colds (66.7%) were more frequent in cattle, but for sheep/goat diarrhea (65%) and sores (55.8%) occurred most commonly. All animal ailments recorded in the present study are common in other communities of Gongshan and northwest Yunnan [10,22].

**Table 2:** Frequency of livestock illness happened in Nu villages, Gongshan County

Type of symptom	Pig (%)	Chicken (%)	Cattle (%)	Sheep/goat (%)
Skin conditions	81.7	--	16.7	16.7
Parasites	68.3	20.0	16.7	35.0
Diarrhea	56.7	66.7	65.0	65.0
Heat, fever, colds	55.8	81.7	66.7	42.5
Sores	42.5	--	--	55.8
Not enthusiastic to eat/refusing food	37.5	--	--	--
Constipation	33.3	--	--	--
Gaseous stomach	14.2	--	--	--
Twitching, shivering, breathlessness	13.3	56.7	42.5	42.5
Lying down all the time	12.5	--	--	--
Retained afterbirth	16.7	--	16.7	20.0
Newcastle disease	--	83.3	--	--
Poison (snake and worm bite)	--	--	30.0	43.3

Due to a great variety of influencing factors in Nu villages, the illness frequency of different animals fluctuates monthly and seasonally. From the data analysis of disease frequency in 2009 (Jan.-Dec.), most animal diseases occurred in the summer and autumn, especially from June to August as temperature and rainfall increased. Many young animals like piglets and chicks died in the spring due to cold weather. Similar results were also found in Gongshan in surveys relating to other ethnic groups' livestock [22,23].

Many villagers were unclear about what caused the various death-by-illness events. Most villagers think that livestock epidemic diseases have become severe only since the 1990 s. Many put this down to increases in wolf populations, which are believed to carry a variety of diseases; increased purchasing of infected pork from local markets; and road improvements which bring more diseases along with larger numbers of tourists. Many villagers also stressed that epidemics spread rapidly because many individuals do not dispose of dead animals properly, causing the spread of diseases by dogs and other animals that come into contact with carcasses (Table 3). In recent years, with variable and unpredictable temperature and rainfall, climate change has been increasingly recognised as a major cause of livestock disease.

**Table 3:** Causes of livestock illness according to respondents

Cause	Frequency
Wolf and dog infection	28
Market infection	38
Outside infection (tourist and animals)	50
Disposal of dead animals	23
Climate change	29
Others	10

### Ethnoveterinary plant species for livestock illnesses

All Nu villagers interviewed in this study used plants to treat animal disease. These treatments were typically made from plant preparations, although other materials were used such as alcohol, human byproducts, gunpowder, and bee skin. Some ethnoveterinary practices have both disease treatment and preventive functions and effects.

**Figure 2:**
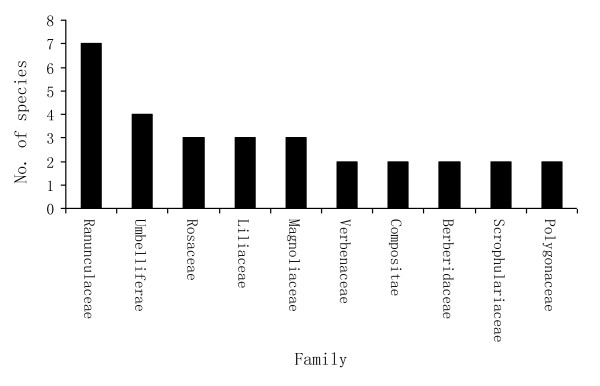
**Distribution of species in different plant families (only families with two or more species shown)**.

Forty five (45) plant species of ethnopharmacological importance were gathered and documented throughout the study period (Table 4). These medicinal plants were distributed among 25 families. The number of species most frequently used by each family was cited as: Ranunculaceae (7), Umbelliferae (4), Magnoliaceae (3), Liliaceae (3), and Rosaceae (3). Other families were represented by at most one species, as shown in figure 2.

**Table 4:** Plant species commonly reported by Nu people for the treatment of livestock diseases in Gongshan County

Scientific name (Latin)	Family	UV	Part used	Disease (or illness)
*Aconitum carmichaeli *Debx.	Ranunculaceae	0.38	Roots	Not enthusiastic to eat/refusing food
*Acorus calamus *L.	Araceae	0.48	Roots	Diarrhea, twitching, shivering, breathlessness
*Agrimonia pilosa *Ldb.	Rosaceae	0.45	Roots	Diarrhea, retained afterbirth
*Allium sativum *L.	Liliaceae	0.52	Bulbs	Witching, shivering, breathlessness, parasites, poison, heat, fever, colds, diarrhea
*Anemone hupehensis *f. alba W. T. Wang	Ranunculaceae	0.33	Rhizomes	Diarrhea, lying down all the time
*Anemone rivularis *Buch.-Ham.	Ranunculaceae	0.33	Roots	Parasites, lying down all the time
*Asparagus setaceus *(Kunth) Jessop	Liliaceae	0.17	Root tubers	Heat, fever, colds
*Berberis wilsonae *Hemsl.	Berberidaceae	0.20	Stems	Diarrhea
*Bupleurum yunnanense *Franch.	Umbelliferae	0.60	Roots and fruits	Heat, fever, colds
*Caltha palustris *L.	Ranunculaceae	0.15	Whole plants	Skin conditions
*Cardamine hirsute *L.	Curiferae	0.37	Whole plant	Diarrhea, gaseous stomach
*Clematis yunnanensis *Franch.	Ranunculaceae	0.38	Stems	Heat, fever, colds
*Clerodendrum bungei *Steud.	Verbenaceae	0.38	Rhizomes	Sores
*Coptis teeta *Wall.	Ranunculaceae	0.52	Rhizomes	Diarrhea, poison
*Davidia involucrata *Baill.	Davidiaceae	0.35	Fruits	Diarrhea, heat, fever, colds
*Epilobium brevifolium *D. Don	Onagraceae	0.43	Roots	Retained afterbirth, sores
*Euphorbia hirta *Linn.	Euphorbiaceae	0.43	Whole plants	Skin conditions, diarrhea
*Foeniculum vulgare *Mill.	Umbelliferae	0.20	Whole plants	Constipation
*Gentiana rhodantha *Franch. ex Hemsl.	Gentianaceae	0.50	Whole plants	Heat, fever, colds
*Hemiphragma heterophyllum *Wall.	Scrophulariaceae	0.35	Whole plants	Retained afterbirth
*Heracleum scabridum *Franch.	Umbelliferae	0.32	Roots	Heat, fever, colds
*Impatiens lecomtei *Hook. f.	Balsaminaceae	0.32	Whole plants	Sores, retained afterbirth
*Kadsura interior *A. C. Smith	Magnoliaceae	0.48	Roots and stems	Retained afterbirth
*Leonurus artemisia (Laur.) *S. Y. Hu	Labiatae	0.53	Whole plants	Retained afterbirth
*Magnolia rostrata *W. W. Smith	Magnoliaceae	0.22	Stems	Twitching, shivering, breathlessness
*Mahonia microphylla *Ying et G. R. Long	Berberidaceae	0.42	Stems and barks	Poison, diarrhea
*Nothopanax delavayi *(Franch.) Harms	Araliaceae	0.20	Roots, stems, barks	Not enthusiastic to eat/refusing food, twitching, shivering, breathlessness
*Picrorhiza scrophulariiflora *Pennell	Scrophulariaceae	0.53	Rhizomes	Heat, fever, colds, parasites, sores
*Plantago depressa *Willd.	Plantaginaceae	0.63	Whole plants	Gaseous stomach, poison, constipation
*Polygonatum kingianum *Coll. et Hemsl.	Liliaceae	0.28	Rhizomes	Gaseous stomach
*Polygonum paleaceum *Wall.	Polygonaceae	0.60	Rhizomes	Heat, fever, colds, retained afterbirth, poison
*Potentilla fulgens *Wall. ex Hook.	Rosaceae	0.35	Roots	Diarrhea, gaseous stomach, constipation
*Ranunculus chinensis *Bunge	Ranunculaceae	0.42	Whole plants	Diarrhea, parasites
*Rubus corchorifolius *L. f.	Rosaceae	0.62	Whole plants	Diarrhea, retained afterbirth
*Rumex nepalensis *Spreng.	Polygonaceae	0.28	Roots and leaves	Diarrhea, constipation, parasites
*Sambucus williamsii *Hance	Caprifoliaceae	0.50	Stems and leaves	Fracture, lying down all the time
*Saussurea costus *(Falc.) Lipech.	Compositae	0.67	Whole plants	Heat, fever, colds
*Schisandra sphaerandra *Stapf	Magnoliaceae	35.0	Fruits	Diarrhea, heat, fever, colds, sores
*Senecio scandens *Buch. -Ham. ex D. Don	Compositae	0.67	Whole plants	Constipation, poison, diarrhea, skin conditions
*Sinodielsia yunnanensis *Wolff	Umbelliferae	0.58	Roots	Retained afterbirth
*Spinacia oleracea *L.	Chenopodiaceae	0.32	Whole plants	Retained afterbirth, sores
*Vaccinium fragile *Franch.	Ericaceae	0.28	Roots	Lying down all the time, parasites
*Verbena officinalis *Linn.	Verbenaceae	0.43	Whole plants	Fracture, retained afterbirth
*Viola yezoensis *Maxim.	Violaceae	0.25	Whole plants	Sores, poison
*Zea mays *L.	Gramineae	0.43	Seeds	Sores

**Figure 3:**
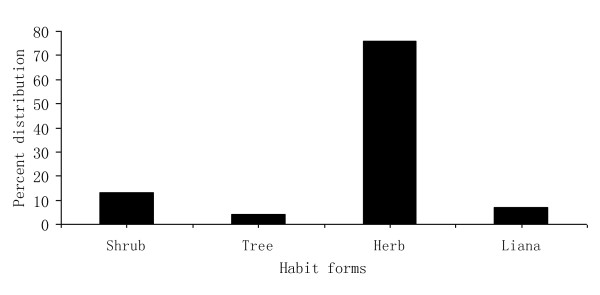
**Percentage distribution of the habit growth forms of medicinal plants in Nu villages, Gongshan County**.

Analysis of the growth forms of these medicinal plants revealed that herbs constituted the largest number or proportion with 32 species (72%), followed by 6 shrubs (13%), 2 trees (4%), and 5 lianas (11%) as shown in figure 3. Most medicinal plant resources (86.7%) were collected wild from alpine grassland and forest areas; only a few (13.3%) were collected from cultivated areas. This indicates that Nu villagers depend on wild sources in the natural environment rather than home gardens to obtain medicinal plants, and medicinal plant growing is poorly developed in the study area. However, in recent years, due to over-exploitation and over-collection by Nu villagers for marketing, these medicinal plants have become more scarce. Though many Nu villagers thought the period of September and November to be the optimal time for medicinal plant collection, they did not reserve a special time to harvest and preserve medicinal plants annually. Rather, they generally looked for and prepared medicinal plants when animals are ill.

Whole plants were the plant parts most frequently used, constituting 36%, followed by roots (22%), fruit/seeds (7%), and mixed plant parts (38%). Such widespread harvesting of whole plants and roots which are important for plant survival threatens the survival and continuity of valuable medicinal plants and has implications for sustainable plant utilization [23,24]. The most important medicinal species were: *Saussurea costus *(Falc.) Lipech. (UV = 0.67), *Senecio scandens *Buch.-Ham.ex D.Don (UV = 0.67), *Plantago depressa *Willd. (UV = 0.63), *Rubus corchorifolius *L. f. (UV = 0.62), *Bupleurum yunnanense *Franch. (UV = 0.60), *Polygonum paleaceum *Wall. (UV = 0.60), *Sinodielsia yunnanensis *Wolff (UV = 0.58), *Picrorhiza scrophulariiflora *Pennell (UV = 0.53), *Leonurus artemisia *(Laur.) S. Y. Hu (UV = 0.53), *Allium sativum *L. (UV = 0.52), *Coptis teeta *Wall (UV = 0.52), *Gentiana rhodantha *Franch. ex Hemsl. (UV = 0.50), and *Sambucus williamsii *Hance (UV = 0.50). Most medicinal plant species being lower UV indicated that there was little consensus on which ethnoveterinary knowledge and remedies were effective in these Nu communities. One reason is that many treatments are unknown to some or many villagers. Moreover, even where a treatment is known to many villagers, they may disagree on its efficacy. This highlights the need for pharmacological evaluation of the species to determine which are efficacious and safe to use [1,2,25-27].

The animal diseases treated with the highest number of ethnoveterinary plant remedies were diarrhea (16 plant species), heat, fever, colds (11 plant species), retained afterbirth (11 plant species), and skin conditions and sores (11 plant species, many sores resulting from skin conditions) (Table 4). However, some plant remedies were rarely used or only for a particular animal type. Plant medicines were processed mostly as mixtures of two or more species, and a few were prepared using one species. Similar to findings of previous research, preparation and application for different types of ailments included decoction, infusion, powder, and crushed remedies [3,24,25]. The most common dosage form is decoction in water, followed by infusion. Previous research performed in NW Yunnan indicated that most plant species used as medicinal treatments were also used as medicinal treatments for both human and animal ailments by other ethnic group communities of NW Yunnan as well as other regions of China [23,28,29]. Unlike some other places [23,30-32], the uses or dosage for human and animals were often imprecise and changeable because there were no traditional experts in Nu villages. Many Nu people confirmed that they did not have accurate dosage standards and just applied dosages for both human and animals according to their past experience.

### Distribution of ethnoveterinary plant remedy knowledge

Almost all ethnoveterinary practices and knowledge of the Nu people are derived from the daily practice of livestock production. There were no publicly recognized traditional experts in ethnoveterinary medicine in the three Nu villages. From the distribution of ethnoveterinary practice knowledge, it was found that women were more likely to know ethnoveterinary practices of pigs and chickens than men. This is consistent with the household division of labour in which women take on more responsibility for feeding and management of pigs and chickens at home [10]. Men knew more ethnoveterinary practices for cattle, sheep and goats than women because men were mostly responsible for the grazing of these animals [10]. Older villagers were more likely to know these practices than all others, but there was no significant difference between young and middle-aged people, suggesting that ethnoveterinary knowledge is still being passed on through its application in the daily practice of livestock raising.

**Figure 4:**
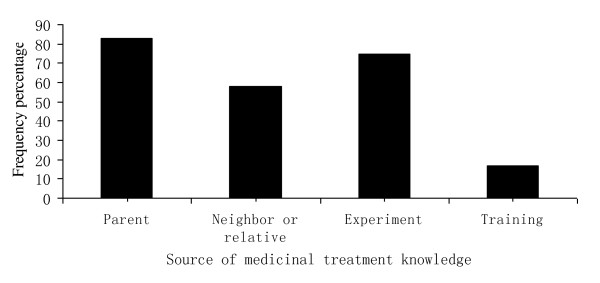
**Source of medicinal treatment knowledge by Nu villagers, Gongshan County**.

Further analysis shows that most medicinal treatment knowledge was from the daily practice of livestock raising, and very little was from training and extension of local animal husbandry agents. Most Nu villagers had learnt about remedies from parents (83%) and neighbors or relatives (58%) (Figure 4). Moreover, based on this knowledge many developed new remedies themselves through practice and experiment (75%). Only a few remedies and knowledge (17%) were learnt from government training.

Like many other ethnic groups around the world [30-38], ethnoveterinary knowledge and practices are very important to Nu culture and livelihoods. However, there are many constraints and difficulties facing the conservation of ethnoveterinary knowledge. Traditional medicine knowledge is difficult to learn and transfer accurately [33,34], and because the practice of traditional medicine is not considered respectable in some rural areas, many are abandoning it [35]. The traditional medicinal knowledge and culture of many ethnic groups is in danger of being lost with the disappearance of biodiversity and negative effects of mainstream culture, processes that are not reversible [7,30,36-39]. Young generations are less willing to inherit traditional professions and learn the precious traditional knowledge handed down by their parents, and because it has been passed down orally from generation to generation it is in danger of extinction [30-33,39-41]. In the present study area, the traditional medicinal knowledge and culture of Nu people is also challenged by western veterinary medicine extension, livelihood change and environment degradation.

### Choice of Treatment Provider

In comparison with modern veterinary medicine, 91.7% Nu Respondents thought the traditional remedies had many advantages: better effects (62.5%), convenience (62.8%) and lower cost (69.9%), and only a few (8.3%) respondents thought ethnoveterinary remedies do not have benefits when compared to modern medicine. All these findings indicate that traditional remedies have high acceptance in Nu villages.

Some factors that influenced Nu villagers' choices of animal disease treatment providers were: availability of local/traditional remedies, distance to providers and indirect time and travel costs, direct costs of medicines and other cash costs, and availability of effective treatments from different providers (Table 5). For most illness conditions, including the most frequent and most severe conditions, Nu villagers preferred to use traditional ethnoveterinary practices. In 2009, 52% of total households interviewed said traditional ethnoveterinary practices were their first choice to treat animal illnesses. In Dimaluo and Qiunatong, a farmer will seek help from the para-vet in the village only if these traditional medicines do not work, or if the farmer does not know any practices relevant to the symptoms. If the para-vets are not available, or if they do not have relevant medicines, some farmers will travel to the town veterinary station 40 kms away and purchase medicines. Because Shuangla is closer to the town veterinary station, if there are no ethnoveterinary practices or if these do not work, villagers tend to go straight to the town veterinary station to purchase medicines. For less severe illness conditions, some villagers will purchase human medicine from a store in the village or from the village doctor.

**Table 5:** The distribution of different treatment providers for animal illness in Nu villages, Gongshan County

Choice of treatment provider	Percentage (%)
Own ethnoveterinary knowledge	51.7
Village para_vets	23.3
Township veterinary service	20.0
Traditional expert	--
Druggist	3.3
Others	1.7

Severity of illness is not a factor influencing farmers' choices. The factors influencing treatment decisions are related to farmers' access to services and to the characteristics of service providers. In the case of Dimaluo and Qiunatong, poverty and distance are considerable factors influencing access to formal providers. Other research has also documented constraints on formal providers to providing services in rural areas, including financial and institutional incentives [39]. The cost and availability of medicines and providers is a factor driving the use of alternative providers, including self-treatment using ethnoveterinary practices and purchasing medicines from human medicine sales points. In this situation, indigenous ethnoveterinary treatments play a very important role in farmers' efforts to maintain animal health.

Research has found that ethnoveterinary treatments are the primary recourse of farmers when their animals are ill. Results of surveys in Gongshan concur with findings elsewhere [25,32,40] that farmers perceive the benefits of ethnoveterinary treatments to be their local availability and low cost [5,6,41-44]. Given the costs associated with seeking formal providers and constraints on formal service provision, ethnoveterinary treatments are often the only available means for Nu villagers to treat ill animals.

## Conclusion

The present study recorded 35 animal ailments and identified major and most common animal diseases, such as skin conditions, diarrhea, heat, fevers, colds, and parasites. Illness frequency among different animal types was compared. Generally, pigs and chickens had the highest number of disease types and disease frequency. One reason is that these animals are more popular than other animals. The other reasons are that there are more disease infection sources from outside such as tourists and local markets dominated by foreign pigs and chickens. Most animal ailments occurred during the summer and autumn because the temperature and rainfall are highest at this time. On the other hand, temperature and rainfall have been variable and unpredictable in recent years, and long drought and heavy rain periods caused skin conditions, heat, fevers, colds, and parasites.

There are 45 plant species used for animal disease treatment in the study area. Most of them are collected from alpine grassland and forest areas and the use and number of them are affected by outside market requirements. Recently, due to over-exploitation and over-collection of wild plant resources these medicinal plants have become increasingly scarce and endangered. Generally the preparation, usage, dosage, and knowledge of these plant medicinal remedies are related to the local social-cultural characteristics of Nu communities. Once animals are ill, most Nu villagers prefer to choose traditional plant remedies, but sometimes their choice is affected by distance to providers and indirect time and travel costs, direct costs of medicines and other cash costs, as well as availability of effective treatments from different providers.

Traditional ethnoveterinary knowledge is related to the local social-cultural characteristics of Nu people and plays a very important role in animal production. This traditional knowledge faces the risk of disappearing due to increasing western veterinary medicine extension, livelihood changes and environment degradation. In order to disseminate and preserve traditional ethnoveterinary knowledge, a number of strategies and actions should be adopted in the future. Firstly, understanding of local traditional knowledge should be increased, especially the local social and religious culture. Secondly, local traditional knowledge and its distribution characteristics should continue to be studied and collected, including traditional knowledge of different ethnic groups. Thirdly, it is necessary to share and disseminate ethnoveterinary knowledge locally, both within and between communities. In some cases, inexpensive allopathic treatments may exist where no effective ethnoveterinary treatments can be verified. Finally, it is important to make ethnoveterinary medicine and knowledge an integrated part of modern animal health care in Gongshan [25].

## Competing interests

The authors declare that they have no competing interests.

## Authors' contributions

All authors contributed equally during the field work, data analysis and preparation of the manuscript, and read and approved the final manuscript.
